# Psychotherapy research for compulsive buying-shopping disorder: Quo vadis?

**DOI:** 10.1016/j.abrep.2025.100591

**Published:** 2025-02-25

**Authors:** Astrid Müller, Patrick Trotzke, Patricia Schaar, Tobias A. Thomas, Ekaterini Georgiadou, Sabine Steins-Loeber

**Affiliations:** a Department of Psychosomatic Medicine and Psychotherapy, Hannover Medical School, Hannover, Germany; b Department of Clinical Psychology and Psychotherapy, Charlotte-Fresenius University of Psychology, Cologne, Germany; c Department of Psychiatry and Psychotherapy, Paracelsus Medical University Nuremberg, Germany; d Department of Clinical Psychology and Psychotherapy, Otto-Friedrich-University of Bamberg, Germany

**Keywords:** compulsive buying, cognitive behavioural therapy, cue reactivity, experimental medicine approach, psychotherapy

## Abstract

Aim The aim of this position paper is to address the question of how psychotherapy research for compulsive buying-shopping disorder (CBSD) should develop further. *Method:* After a brief summary of existing psychotherapy research, this paper concentrates on the advantages and shortcomings of previous psychotherapy studies and offers recommendations for future psychotherapy research in the domain of CBSD. *Results:* Systematic reviews indicate that cognitive behavioural therapy is the most researched form of psychotherapy and presents a helpful intervention for reducing the symptom severity of CBSD. Notwithstanding the positive outcomes, the psychotherapy studies to date are limited by methodological shortcomings, which reduce their validity and generalizability. While research into the psychological mechanisms of offline and online CBSD has expanded considerably, psychotherapy research has not kept pace with this growth. Although the majority of individuals with CBSD engage in online shopping, the problematic usage of shopping websites has not been considered in any of the treatment studies to date. *Conclusion:* The application of the experimental medicine framework to psychotherapy research for CBSD may enhance the integration of findings on psychological mechanisms of CBSD with existing treatment concepts for CBSD. Moreover, it is necessary to consider the impact of technological factors and e-marketing in the context of treatment. There is a need for 1) proof-of-concept studies to test specific interventions that target specific psychological processes and mechanisms of CBSD, and 2) high-quality psychotherapy studies to test the efficacy and effectiveness of new treatment approaches in accordance with the scientific standards for randomised controlled trials

## Highlights

• Cognitive behavioral therapy is the most researched psychotherapy for CBSD.
• Psychotherapy studies to date are limited by methodological shortcomings.
• Findings on psychological mechanisms should be integrated with existing treatments.
• Adopting the experimental medicine approach can advance psychotherapy research.
• More proof-of-concept and high-quality psychotherapy studies are needed.

## 1 Introduction

Compulsive buying-shopping disorder (CBSD) has only recently appeared as an example of other specified impulse control disorders in the ICD-11 (WHO, 2023). However, CBSD is not a new phenomenon, as it was first described over a century ago (Demling and Müller, 2023, Kraepelin, 1899) and has an estimated prevalence of approximately 5% (Maraz et al., 2016). In the late 1980, case reports of insight-oriented (Krueger, 1988, Winestine, 1985) and cue-exposure therapy (Bernik et al., 1996) have shown promise, prompting the initiation of controlled treatment trials. The cognitive behavioural therapy (CBT) trial conducted by Mitchell and colleagues (2006) can be regarded as the first controlled psychotherapy study for CBSD.

Since then, research into CBSD has expanded considerably, with studies now encompassing not only phenomenology and psychosocial aspects (e.g., comorbidity, prevalence), but also psychological and neurobiological processes that may be involved in the development and maintenance of CBSD (Black, 2022, Kyrios et al., 2018, Lörsch et al., 2025, Müller et al., 2019, Otero-Lopez, 2022, Thomas et al., 2023, Thomas et al., 2024). Many studies have identified parallels between CBSD and other addictions, including cue reactivity and craving, seemingly compulsive motivations and habitual behaviours, and diminished control over the activity (Brand et al., 2025, Kyrios et al., 2018, Starcke et al., 2018, Thomas et al., 2023, Trotzke et al., 2021), supporting its classification as a disorder due to addictive behaviours (Brand et al., 2022). Unfortunately, psychotherapy research is not keeping pace with laboratory-based experimental research. No new controlled psychotherapy studies have been published for over a decade, and - at least according to the available literature - there seems to be a lack of recent innovative developments in the psychotherapy of CBSD (cf. Goslar et al., 2020, Müller et al., 2023, Vasiliu, 2022).

The objective of this position paper is to address the question of how psychotherapy research for CBSD could and should develop further. We start with a brief summary of the current state of psychotherapy research in the field of CBSD, without providing a systematic review, as this has recently been done elsewhere (Goslar et al., 2020, Müller et al., 2023, Vasiliu, 2022). The subsequent section provides a critical view on the advantages and disadvantages of previous psychotherapy research. Finally, we exemplify suggestions for future psychotherapy research for CBSD.

## 2 Psychotherapy research for CBSD – a brief summary

At present, CBT is the most researched form of psychotherapy for CBSD. Systematic reviews indicate that, in particular, face-to-face group CBT is a helpful intervention for reducing the symptom severity of CBSD when compared to a waitlist control condition (Goslar et al., 2020, Hague et al., 2016, Leite et al., 2014, Müller et al., 2023, Vasiliu, 2022). The favourable outcomes associated with group CBT were corroborated by an open-label study that employed individual CBT for CBSD in a relatively large clinical sample (*N*=97) (Granero et al., 2017).

In our opinion, it is important to emphasize that the controlled psychotherapy studies conducted to date were carried out with great enthusiasm, though probably often not sufficiently funded and limited by methodological shortcomings (Benson et al., 2014, Mitchell et al., 2006, Müller et al., 2013, Müller et al., 2008). Notwithstanding the positive results, there are concerns and open questions that reduce the validity and generalizability of the outcomes.

## 3 Psychotherapy research for CBSD - mind the gap

The methodological limitations of previous psychotherapy studies include the relatively small sample sizes, the use of a wait-list control condition rather than alternative active treatments, and the pre-post design rather than multiple-measure approaches to test treatment effects. The CBT studies were all manual-based. While this appears to be a methodological strength, it is important to bear in mind that psychotherapists’ adherence to the manual was not examined in any of the published studies, which in turn presents a methodological weakness. To the best of our knowledge, third-wave CBT or psychotherapy approaches other than CBT have not been subjected to a systematic investigation, particularly not using a controlled design. Apart from a small number of case reports (e.g., Krueger, 1988, Winestine, 1985), no studies have been published on psychodynamic approaches. This is surprising, given that CBSD episodes could be conceptualised as dysfunctional external behaviour that arises from the escape from dysregulated negative affect (Gori et al., 2024). Many of our patients present with a low level of structural integration and insecure attachment to consumer goods (Norberg et al., 2018). They use excessive consumption as a way to deal with alexithymia, narcissistic needs, materialism, dysregulated negative internal states or identity confusion (Brand and Müller, 2023, Claes et al., 2016, Gori et al., 2024, Moulding et al., 2017, Moulding et al., 2021). Although partnership and familial conflicts have the potential to facilitate the development of CBSD, or to maintain it in the long term (Schäfer et al., 2019), there has been no systematic investigation of the potential of couple-based or systemic therapy approaches as used e.g. in the treatment of depressive disorders or obsessive-compulsive disorder (Whisman & Baucom, 2012).

Despite the large body of literature on the significance of mental comorbidity (e.g., anxiety, depression, compulsive hoarding of consumer goods, binge eating) (Brand and Müller, 2023, Fernandez-Aranda et al., 2008, Frost et al., 2011, Kim et al., 2018, Mitchell et al., 2002, Müller et al., 2010), temperament and personality features (Claes and Müller, 2017, Granero et al., 2016, Otero-Lopez, 2022, Otero-Lopez and Villardefrancos, 2013) in CBSD, these aspects have been largely overlooked in psychotherapy studies to date, with a few exceptions (Granero et al., 2017, Mestre-Bach et al., 2016, Müller et al., 2008). Also, influences of patients’ stress vulnerability (Thomas et al., 2024) and maladaptive beliefs about purchasing and shopping (Fioravanti et al., 2025) were not considered in psychotherapy research. Finally, there has been a lack of consideration of specific therapeutic factors, non-specific therapeutic factors (e.g., patient engagement, therapeutic alliance, group cohesion), the contextual and cultural determinants of CBSD in psychotherapy research.

While the list of limitations may seem very extensive, it is not exhaustive. It is our intention to clarify that the objective of this position paper is not to provide a litany of complaints or fundamental criticism of previous CBSD psychotherapy research. Some of us were involved in the studies whose methodological weaknesses we have mentioned. As previously stated, these studies constitute pioneering work in the field of CBSD. However, given the progress of research on CBSD, the lack of studies investigating the mechanisms of change is hard to understand. There is not only a great need for high-quality treatment studies (in the sense of RCTs) that overcome the methodological weaknesses of previous trials, but also for proof-of-concept studies that address the mechanisms of change. In other words, it is time to merge recent findings on psychological and neurobiological processes and mechanisms with existing treatment concepts for CBSD (Brand and Potenza, 2021, Müller et al., 2023, Young and Brand, 2017). In the following, we put forward a few suggestions for the potential enhancement of psychotherapy research in the field of CBSD.

## 4 Psychotherapy research for CBSD - implications for future studies

As outlined above, there is a broad range of variables that were not considered sufficiently in previous psychotherapy studies for CBSD (and we have by no means mentioned them all). However, rather than attempting to address all possible factors that may be important in the psychotherapy of CBSD or may impact the outcome, at present, we propose to focus on those psychological processes for which empirical evidence exists that they contribute to the development and maintenance of CBSD based on the literature. In this regard, it can be beneficial to consider the application of the experimental medicine framework to CBSD psychotherapy research.

### 4.1 The application of the experimental medicine approach

The experimental medicine framework (Riddle & Science of Behavior Change Working, 2015; Sheeran et al., 2017) may help bridging the gap between basic research (focusing on psychological processes and mechanisms) and psychotherapy research that - so far - has primarily addressed the question whether psychotherapy works but not how it works. The idea is not new at all in psychotherapy research. For example, Eysenck called for cooperation between experimental psychology and applied behavior therapy as follows: “To effect a reunion of two disparate disciplines like that must be one of the aims of behavioral therapists.” (Eysenck, 1973, p. 26).

The experimental medicine approach utilizes mechanism-focused methods, thereby testing the relevance of putative intervention targets believed to be responsible for behavioural change within four steps: 1) identification of an intervention target (path A), 2) development of assays/measures to validate the target (path B), 3) engaging the target through experimental interventions (path C), and 4) testing the degree to which target engagement results in the desired behaviour change (path D) (Nielsen et al., 2018; Riddle & Science of Behavior Change Working, 2015; Sheeran et al., 2017). This approach has already been suggested for other psychological interventions, e.g., the translation of laboratory findings regarding food and alcohol consumption into therapeutic interventions (Field et al., 2021) or to identify novel determinants of addiction (Bickel et al., 2019). The strategy could also help to achieve a better understanding of how and why certain manipulations impact core psychological processes and mechanisms in CBSD or not, thus validating specific target engagement.

**Fig. 1:**
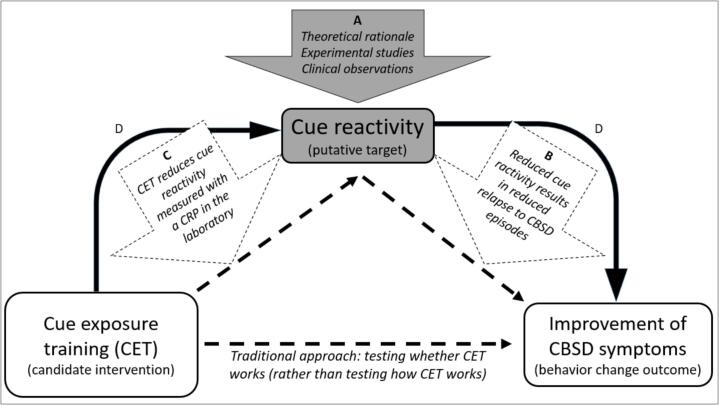
An exemplary application of the experimental medicine approach to CBSD (Figure adapted from Riddle, 2015, Sheeran et al., 2017, Field et al., 2021) CRP = cue reactivity paradigm, CBSD = compulsive buying-shopping disorder

Here we apply the experimental medicine approach to CBSD, focusing on cue reactivity as an example of a putative target, and computerized cue exposure training as an example of a candidate intervention to modify this target (see Fig. 1). In this particular instance, the research question to be addressed reads as follows: Does a computerized cue exposure training reduce cue-induced cravings towards shopping-related cues and do changes in cue reactivity result in a decrease of CBSD symptom severity? Research and clinical observations identified cue reactivity as a relevant psychological process in CBSD (Müller et al., 2025, Starcke et al., 2018, Thomas et al., 2023, Trotzke et al., 2019, Trotzke et al., 2021, Vogel et al., 2019, Voth et al., 2014). Hence, the first of the four steps of the experimental medicine approach has already been taken (path A) and cue reactivity may be defined as an important target for interventions aiming to reduce CBSD behaviour. The second step is to validate the putative target (i.e. cue reactivity) by examining when, how and to what extent it leads to behaviour change (path B). Self-report measures and/or clinical interviews or functional analyses could be used to examine if cue-induced craving triggers shopping episodes and/or elicits relapses into CBSD episodes and if reduced craving responses are associated with less shopping episodes or a lower risk of relapses. In the third step, cue reactivity could be experimentally targeted, e.g., by using a computerized cue exposure training (CET). The training effect could be measured with a cue reactivity paradigm (assessing affective responses toward visual shopping-related cues, e.g., the urge to buy) (path C). Path C may also include subsequent laboratory studies to test different samples (e.g., with diagnosed CBSD vs. risky shopping) and settings (e.g., virtual reality exposure, imaginal exposure, in vivo exposure), to examine potential dose-response effects (e.g., varying frequency and/or duration of the training) and to investigate the influence of different cues (e.g., distal vs. proximal shopping cues, individualized vs. generic shopping cues). Path C could, for example, include a pilot study where participants with diagnosed online CBSD are exposed in vivo to their favourite internet shopping application and instructed to direct their attention to the conditioned urge to browse that arises, but prevent browsing on the shopping site. They should resist the urge to browse until they notice its significant reduction. Similar CET approaches have shown to be helpful in preventing relapses in substance use disorders (Kiyak et al., 2023) or gambling disorder (Bergeron et al., 2022). The results of paths A, B, and C could be utilized to augment face-to-face group CBT with individual computerized cue exposure sessions. The effect of the cue exposure training could then be determined in a randomized controlled trial comparing the blended CBT with the traditional group CBT (path D).

### 4.2 Addressing both cognitive and affective networks

As previously stated, CBT has been demonstrated to be helpful in reducing CBSD symptoms. However, not all patients are able to completely normalize their shopping behaviour over the course of psychotherapy or maintain improvements in CBSD symptoms over the long run. An unpublished follow-up study (Steiger, 2010) of individuals who had undergone group CBT for CBSD three to five years ago (Müller et al., 2008) indicated that six of the 26 participants exhibited symptoms of CBSD and scored above the threshold for pathological buying on the shopping version of the Yale-Brown Compulsive Obsessive Scale (Monahan et al., 1996). In line with the preceding suggestions, we assume that patients who regularly relapse would benefit from complementary or other interventions than traditional group-CBT alone. Viewing through the lens of dual-process models in decision-making with regard to addictions (Bechara, 2005), CBSD episodes - including relapses during and following CBT - may be explained by the imbalance between two interacting neural systems: the impulsive and the reflective system (for details see Trotzke et al., 2017). CBT interventions include cognitive techniques (e.g., identifying automatic thoughts, cognitive restructuring) that focus primarily on the reflective system, specifically the cognitive networks of the dorsolateral prefrontal cortex (dlPFC). Considering the imbalance hypothesis, the treatment of CBSD should also consist of interventions that target the impulsive system (affective networks; e.g., ventromedial prefrontal cortex, vmPFC) to a greater extent than conventional CBT does (Trotzke et al., 2017). There exist several psychotherapeutic concepts that focus on affective and cognitive processes (and their interaction) which could certainly be beneficial in improving emotion regulation and decision-making abilities (e.g., CET, mindfulness-based interventions, eye movement desensitization and reprocessing). Own clinical work and discussions with other experts in the field show that some of these interventions are already being used in routine clinical practice. To the best of our knowledge, however, the utilisation of these interventions in individuals with CBSD has not been the subject of a systematic investigation. We would therefore like to share some thoughts that could be taken into account in future psychotherapy research. The following statements are not intended to be an exhaustive list of interventions that may be used to address emotional networks, but rather possible examples of research topics.

In the preceding paragraph, the experimental medicine approach was introduced, with the example of cue reactivity as the putative intervention target and CET as the candidate intervention. In the same manner, the application of mindfulness-based techniques (candidate intervention) to modify cue reactivity (putative target) should be systematically investigated. Mindfulness refers to purposeful awareness that arises from non-judgementally paying attention to internal and external experiences in the present moment (Kabat-Zinn, 2005). Mindfulness-based interventions are assumed to be effective in managing craving, negative affective states (e.g., discomfort, distress) and habitual behaviour by modulating cognitive, affective, and psychophysiological processes inherent to self-regulation and reward processing (for details see Garland & Howard, 2018). The implementation of mindfulness-based programs has been shown to reduce cravings and relapses in addictive behaviours (Bowen et al., 2021, Brandtner et al., 2022, Fendel et al., 2024, Garland and Howard, 2018, Goldberg et al., 2018, Lomas, 2024, Zgierska et al., 2009). Mindful attention for potentially triggering affective states or challenging external situations could help individuals with CBSD to cope better with physical and emotional discomfort and to break the destructive cycle of relapse (Bowen et al., 2021). This therapeutic approach should therefore be systematically investigated in relation to CBSD.

Eye Movement Desensitization and Reprocessing (EMDR) therapy is a trauma-focused treatment for post-traumatic stress disorder that uses a dual attention paradigm, involving rapid bilateral eye movements while recalling traumatic memories, thereby neutralizing their emotional charge and facilitating appropriate processing within working memory (Solomon & Shapiro, 2008). EMDR therapy may be a promising approach for addressing memory representations of cue-induced cravings in addictions, as it has been shown to facilitate the adaptive processing and integration of these memory traces (Hase et al., 2008). Using bilateral eye movements has demonstrated efficacy in desensitizing memories and imagery-based desire thinking and cravings in the context of substance-related and behavioral addictions (Brandtner and Brand, 2021, Littel et al., 2016, van Minnen et al., 2020). In light of these encouraging results and considering the well-known connections between traumatic childhood experiences and CBSD (Imperatori et al., 2023, Sansone et al., 2013), the application of EMDR techniques as candidate interventions to target cue reactivity in CBSD should be examined in future proof-of-concept studies.

### 4.3 Considering technological factors that may drive online CBSD

The majority of individuals with CBSD engage in online browsing and purchasing activities. Hence, it seems important to consider environmental and technological factors that may drive addictive usage of shopping websites beyond psychological processes involved in CBSD (Brand, 2022, Flayelle et al., 2023). From our perspective, the rise of e-commerce platforms, social e-marketing and intertwined internet applications should not be trivialised. This remark pertains particularly to the seamless convergence of shopping and social network sites, or the monetization of videogames. Social network users may become preoccupied with online shopping via frequent exposure to influencer posts, pop up advertisements or links to shopping websites (Müller et al., 2022, Sharif et al., 2021, Wegmann et al., 2023). Likewise, problematic online shopping and video gaming are often related (Greenberg et al., 2022). Uncontrolled in-game-purchases and microtransactions can add up very quickly and result in large debts (King et al., 2019). In this context, it may be beneficial to use ecological momentary interventions to deliver prevention or treatment modules in real time (Balaskas et al., 2021, Dao et al., 2021). It certainly makes sense to reach individuals at-risk for CBSD, or those already holding a CBSD diagnosis, where they browse and shop, namely on the internet. Online-based motivational interventions have been successfully applied to individuals with risky or pathological internet use (Dieris-Hirche et al., 2023). Likewise, ecological momentary interventions have been shown to be effective in reducing risky alcohol consumption (Balaskas et al., 2021), cannabis use (Beckham et al., 2024) and gambling (Merkouris et al., 2020). These treatment approaches could also be beneficial in the context of online CBSD. Further research is warranted to investigate such online-based momentary interventions.

## 5 Conclusions

It is now time to make further progress in the psychotherapy research for CBSD and pursue scientifically sound, innovative psychotherapy approaches that work. The inclusion of CBSD in the ICD-11, albeit not yet as a standalone mental disorder, and the encouraging increase in research may contribute to this.

Answering the question what general lessons we can draw from our critical view on existing psychotherapy research, we finally want direct attention to the following key conclusion. We should enhance our understanding of how to modify putative intervention targets and to ascertain which interventions are effective in promoting the desired behavioural change in individuals with CBSD. Moreover, we should gain knowledge concerning the individuals for whom the interventions are helpful, and the circumstances under which they work. There is a need for proof-of-concept studies testing specific interventions that target specific psychological processes and mechanisms of CBSD and for subsequent high-quality psychotherapy studies to test the efficacy and the effectiveness of new treatment approaches in accordance with the scientific standards for RCTs, which requires sufficient third-party funding.

## CRediT authorship contribution statement

**Astrid Müller:** Writing – review & editing, Writing – original draft, Conceptualization. **Patrick Trotzke:** Writing – review & editing, Supervision. **Patricia Schaar:** Writing – review & editing. **Tobias A. Thomas:** Writing – review & editing. **Ekaterini Georgiadou:** Writing – review & editing. **Sabine Steins-Loeber:** Writing – review & editing, Conceptualization.

## Declaration of competing interest

The authors declare that they have no known competing financial interests or personal relationships that could have appeared to influence the work reported in this paper.

## Data Availability

No data was used for the research described in the article.
